# Diffusion Model of Preemptive-Resume Priority Systems and Its Application to Performance Evaluation of SDN Switches

**DOI:** 10.3390/s21155042

**Published:** 2021-07-26

**Authors:** Tomasz Nycz, Tadeusz Czachórski, Monika Nycz

**Affiliations:** 1 Department of Distributed Systems and Informatic Devices, Silesian University of Technology, 44-100 Gliwice, Poland; tomasz.nycz@polsl.pl; 2 Institute of Theoretical and Applied Informatics, Polish Academy of Sciences, Bałtycka 5, 44-100 Gliwice, Poland; 3 Department of Computer Networks and Systems, Silesian University of Technology, 44-100 Gliwice, Poland; monika.nycz@polsl.pl

**Keywords:** diffusion approximation, transient states, SDN, priority queues

## Abstract

The increasing use of Software-Defined Networks brings the need for their performance analysis and detailed analytical and numerical models of them. The primary element of such research is a model of a SDN switch. This model should take into account non-Poisson traffic and general distributions of service times. Because of frequent changes in SDN flows, it should also analyze transient states of the queues. The method of diffusion approximation can meet these requirements. We present here a diffusion approximation of priority queues and apply it to build a more detailed model of SDN switch where packets returned by the central controller have higher priority than other packets.

## 1. Introduction and an Overview of Existing Results

Software-Defined Networking (SDN), flexible in service of various applications, becomes an alternative to the classical Internet. Traffic and its routing are supervised here by a programmable central controller; its frequent decisions adapt the routing to the current load observed in the network switches, aiming to avoid their congestion. The controller may also activate and deactivate switches to save energy. The network supports multiple classes of traffic having different statistical behavior with different QoS requirements. The service differentiation and QoS provisioning techniques may lead to non-stationarity in the overall traffic of the network. Therefore, traffic intensity in SDN switches is frequently changing. It is recommended that an investigation of performance based on queueing models should allow the transient analysis of packet queues in the switches.

SDN is already an advanced technique. The article [1] presents the history and evolution of programmable networks starting from telephone networks, through packet networks, then Internet, and finally to SDN networks over almost 50 years. An overview of the SDN network, its scalability, elasticity, reliability, and availability are shown in [2]. Reference [3] discusses SDN performance within a data center. Improvements are classified following data, control, and application planes and network type: cloud, wireless and wide-area. The article [4] reviews SND’s significant benefits and possible applications. A comparison of various SDN programming languages, such as Flow-based Management Language, then Nettle, Procera, Frenetic, Netcore, Frenetic-OCaml, Pyretic, and NetKAT, is given in [5]. Paper [6] presents an industry survey conducted among IT professionals on network virtualization and SDN within cloud computing, discussing its scalability and roadmap.

Several papers focus on control and data planes issues of SDN controllers; e.g., Reference [7] addresses the problem of logical consistency within data plane nodes when policy rules within data plane nodes are not synchronized with the SDN controller because of network delays, especially within distributed, hierarchical, or flat control planes. The increase in the probability of desynchronization leads to frequent erroneous packet handling or network failure; the article defines requirements that should be met to limit consistency problems. The article [8] examines the validity of SDN dogma, saying that all control should be moved from the data plane to the SDN controller. It may be incorrect for network functions that require only a local view. Middleboxes performing such operations should stay in the data plane to prevent asking a remote SDN controller about local network information; however, their location and rules should be defined in the control plane. The proper selection of controllers within the control plane is reviewed in [9]. Controllers are divided into two groups: centralized (such as beacon, maestro, meridian, or rosemary) and distributed (such as fleet, hyperflow, onix, or smartlight). Additionally, the paper compares the throughput and latencies of selected controllers. The article [10] analyses the usage of SDN mechanisms in the context of wide-area networks; the main focus is on the distribution of SDN controllers within control planes and load balancing, fault tolerance, and monitoring of network nodes within the data plane. The problem of proper placement of SDN controllers is surveyed in [11]. The goal should be achieved by defining the number of required controllers, their location within the network, and their mapping with data-plane nodes based on many network parameters such as latency, resilience, QoS, or general network objectives. Investigated methodologies are divided into two groups, one looking for optimal solutions and the other for heuristic sub-optimal solutions. The problem of proper placement of controllers is also examined in [12]. The authors compare results obtained by different classes of algorithms, such as clustering, integer linear and quadratic programming, evolutionary bio-based, genetic, heuristic, greedy, and simulated annealing based on requirements such as latency, load balancing, fault tolerance, the optimal number of controllers, cost, and control plane communication. In [13], the authors divided existing solutions for controller placement problems into “capacitated” and “uncapacitated” categories. Both categories aim to decrease the number of controller failures; however, the second one does not consider the controller’s load and capacity during its placement as a constraint.

Papers concentrating on traffic within SDN networks are surveyed in [14], which also describe strategies that aim to lower the latency within the network. The first strategy involves traffic identification and prediction; the second is based on congestion control; the next one concentrates on load balancing, and the other investigates flow table management. Additionally, edge computing and virtualization are taken into account. The mechanisms of packet forwarding within the SDN network are investigated in [15]. Specifically, forwarding table entries in SDN nodes are described and classified, considering wildcard rules, their priority, validity, placement within multiple tables, and integration of traffic statistics. A review of methods used to predict future traffic and congestion is shown in [16]. The prediction is based on historical and current real-time traffic data. The load balancing and energy-efficient routing within the SDN network are analyzed in [17]. Additionally, solutions for fault-tolerant controller placement problems and end-to-end security challenges are presented. A more detailed review, [18], investigates the problem of green computing concerning SDN networking. The authors present the Green-SDN taxonomy and its detailed analysis and propose a framework to increase its security and efficiency while maintaining reduced energy consumption and environmental impact.

The comparison of SDN architecture with a traditional system is presented in [19]. This article lists the drawbacks of traditional systems and describes SDN networks’ management and their further research challenges. The work of [20] analyzes different SDN control plane architectures: centralized, distributed, and hybrid. The comparison is based on numerous factors such as scalability, consistency, reliability, interoperability, controller placement, load balancing, security, etc. Hybrid SDN network architectures are examined in [21]. The approach is a mixture of centralized and decentralized paradigms, e.g., traditional distributed routing algorithms and SDN control plane routing. The article classifies different hybrid SDN models, describes their advantages and disadvantages, and compares them. The article [22] focuses on hybrid SDN architecture; in addition to classifying hybrid models, it presents additional topics such as security and privacy issues, network management with telemetry support, fault tolerance, load balancing, and quality of service. QoS within SDN networks is investigated in [23]; the authors analyze the influence of scalability, consistency, reliability, and load balancing on QoS. Additionally, it examines challenges stemming from interactions between controllers and switches, standards for communication within the control plane and controllers’ placement, managing traffic load, and security.

Queueing theory has supported the process of design and performance evaluation of communication systems since the beginning of telephony and telegraphy, that is, since the times of Erlang and Engset. It has great potential to be used in investigations in problems mentioned above and has already been applied in some analyses of SDN performance. The models used until now are mainly elementary queueing systems, i.e., M/M/1 stations with Poisson arrivals and exponential service time distribution, representing switches and controllers, e.g., in [24] Jackson’s network model, an open network of M/M/1 queues is used for this purpose. Furthermore, Reference [25] presents a tool for SDN network visualization and performance prediction based on M/M/1 models and shows its results with the use of actual data. In [26], a queueing model is refined by the use M/Geo/1 model where service times are geometrically distributed. A more general model of switch and controller is presented in [27,28]. It is based on a preemption-based packet-scheduling priority scheme where a higher priority is assigned to packets routed to switch from the controller. The solution is approximative; the authors present a method to decompose the system with priority and non-priority queues into two systems with one type of queue. In [29], a Markov model of the switch with preemptive priority and non-priority queues and controller, based on a Markov chain, is presented. The interarrival time distribution is composed of multiple phases, so the model is close to the G/M/1/N station. A similar model with general input and non-preemptive priority was proposed in [30]. A recent article [31] presents a model where the switch is represented by three M/M/n/m stations in series, and the controller is modeled by two similar stations, representing the modules of both devices—switch and controller exchange packets with fixed probabilities. A few other models were based on deterministic or stochastic network calculus [32,33,34].

All above models have two deficiencies coming from limitations of queueing theory: (i) they are based on the assumption that the network is in a steady state—i.e., the flows are stable, and network metrics such as queueing delays, the length of packet queues in buffers of SDN switches, and packet losses do not depend on time; (ii) they assume exponential interarrival and service time distributions. This is not valid in the case of SDN. QoS-driven routing creates time-dependent traffic and variable network topology, and it is important to understand the behavior of SDN switches affected by sudden changes of paths and routing made by the SDN controller. The flows are not Poisson, and the service times are not exponentially distributed. The sudden changes of flows have performance consequences, including queueing delays and packet losses, which can only be understood via time-dependent transient analysis. However, conventional queueing network theory is poorly adapted to transient analysis. Even in the case of the simplest single-server system, i.e., M/M/1 queue, the transient solution leads to the use of Bessel function expansions; see [35] for infinite and [36,37] for finite M/M/1/N queues. Some particular cases referring to transient queues were analyzed in [38,39,40]. It is even harder model interconnected systems in transient cases.

Transient behavior of the switch-controller tandem is considered recently in [41]. The authors analyze traffic recorded at a virtual SDN network (mininet). Using a statistical test, they find that it is not stationary. Therefore, they use an approximate transient approach, Pointwise Stationary Fluid Flow Approximation. The balance of input and output flows, taken together with steady-state formulas for M/M/1 and G/M/1 stations, defines the time-dependent evolution of mean queues of switch and controller. It is assumed that the average number of packets at steady state is equal to the average number of packets in non-stationary queue at equilibrium point [42]. Such a model is approximate and limited to mean values (but not distributions) of queues and delays, and it cannot give us, e.g., loss probabilities.

In general, to model stations in a transient regime, the choice of the method includes numerical solution of Markov models, fluid flow approximation, e.g., Reference [43], and diffusion approximation [44]. In Markov models solved numerically, the interarrival and service time distributions may be represented by a system of exponentially distributed phases and fitted to any distribution. Special tools can do this automatically, e.g., Reference [45]. However, this approach is bounded by state explosion; the number of the differential equations (one equation per one state of the model) becomes intractable. Fluid flow approximation, e.g., Reference. [46], similar to the approach presented in [41], may be applied to large topologies. However, it is less exact than the third approach, which is diffusion approximation. We opt for the latter method as it combines transient solutions with the possibilities of including general distributions into the model, and its results are in the form of distributions, not only mean values.

Recently, we have already applied diffusion approximation in modeling a single SDN switch [47] and a network of switches [48]. These models represent an SDN switch as a G/G/1/N station, disregarding communication between the switch and the controller. Here, we develop a diffusion model of a priority station using ideas we proposed in [49], test its quality, and apply it to investigate the communication between the switch and controller. When the flow of an arriving to the switch packet is not identified (it does not exist in the table of flows of the switch), the packet is sent through the uplink channel to the controller to decide on its routing. Then, it returns to the switch with information on its itinerary and is served on a priority basis. Except for the use of the same method, the models and results in [47,48] and here are different. The extension of the presented model into a more complex system of switches and controllers is straightforward.

The rest of the article is organized as follows. Section 2 presents the known diffusion model of a single FIFO station and proposes a new one with priority queues. Section 3 investigates the quality of the priority model using numerical examples, Section 4 presents the rules of a network model composed of single-station models, Section 5 presents an example where diffusion models are implemented to analyze the performance of SDN switch and its communications with the SDN controller, and conclusions are presented in Section 6.

## 2. Diffusion Single Station Models

### 2.1. First-In-First-Out G/G/1/N Station 

With this method, proposed in [44], the distribution of the number of queued packets in the buffer is represented by the density function of a diffusion process.

The idea comes from the observation that the queue N(t)—a discrete stochastic process—and the diffusion proces X(t)—a continuous stochastic process—both have normally distributed changes. For any distribution A(x) of interarrival times, with mean 1/λ and variance $\sigma_{A}^{2}$, the number of arrivals during an interval Δ tends to the normal distribution with mean λΔ and variance $\sigma_{A}^{2}\lambda^{3}$. For any distribution B(x) of service times with mean 1/μ and variance $\sigma_{B}^{2}$, the number of completed services during Δ tends to the normal distribution with mean μΔ and variance $\sigma_{B}^{2}\mu^{3}\Delta$. Therefore, after the interval Δ, the changes in the number of customers present in the queue are subject to the normal distribution with (λ−μ)Δ and variance $(\sigma_{A}^{2}\lambda^{3}+\sigma_{B}^{2}\mu^{3})\Delta$.

The diffusion process with density function, if unrestricted, given by Equation (1)

(1) $$\frac{\partial f(x,t;x_{0})}{\partial t}=\frac{\alpha }{2}\frac{\partial^{2}f\left(x,t;x_{0}\right)}{\partial x^{2}}-\beta \frac{\partial f(x,t;x_{0})}{\partial x}\;,$$

has normally distributed changes in δt with mean βt and variance αdt; therefore the choice of these parameters

(2) $$\alpha =\left(\sigma_{A}^{2}\lambda^{3}+\sigma_{B}^{2}\mu^{3}\right)=C_{A}^{2}\lambda +C_{B}^{2}\mu ,\;\beta =\lambda -\mu$$

where $C_{A}^{2}=\sigma_{A}^{2}\lambda^{2}$ and $C_{B}^{2}=\sigma_{B}^{2}\mu^{2}$ are the square coefficients of variation of A(x), B(x) distributions, enhances similarity of N(t) and X(t).

The diffusion process should be constrained by barriers, following the limitations of a real queue: one barrier is placed at x=0 and the other (if the queue size is limited to *N* customers) at x=N; X(t)=0 means that the queue is empty at time *t* (idle period of the station), and X(t)=N means that the queue is saturated and the arriving customers are rejected (saturation period). We assume that they correspond to interarrival and service times, but in fact these are rather their residual lifetimes; e.g., the idle time is not the interarrival time but the time between the moments when the last customer in the previous busy period left the system and the first in the next busy period came. Following [44], we assume that the process after a stay at x=0 jumps to x=1 with intensity λ (arrival of a first customer in the new busy period) and jumps from *N* to N−1 with intensity μ (departure of a customer de-blocking the queue). In this case,

(3) $$\begin{array}{ccc} \frac{\partial f(x,t;x_{0})}{\partial t} & = & \frac{\alpha }{2}\frac{\partial^{2}f\left(x,t;x_{0}\right)}{\partial x^{2}}-\beta \frac{\partial f(x,t;x_{0})}{\partial x}\\ & + & \lambda_{0}p_{0}\left(t\right)\delta \left(x-1\right)+\mu_{N}p_{N}\left(t\right)\delta \left(x-N+1\right), \end{array}$$

$p_{0}\left(t\right)$ and $p_{N}\left(t\right)$ denote the probabilities that the process is at a barrier at time *t*, and their terms refer to the jumps from barriers. The probabilities of being in the barriers are defined by additional balance equations:

(4) $$\frac{dp_{0}\left(t\right)}{dt}=\lim_{x\to 0}\left[\frac{\alpha }{2}\frac{\partial f(x,t;x_{0})}{\partial x}-\beta f\left(x,t;x_{0}\right)\right]-\lambda_{0}p_{0}\left(t\right),$$

(5) $$\frac{dp_{N}\left(t\right)}{dt}=-\lim_{x\to N}\left[\frac{\alpha }{2}\frac{\partial f(x,t;x_{0})}{\partial x}-\beta f\left(x,t;x_{0}\right)\right]-\mu_{N}p_{N}\left(t\right).$$

The steady-state solution of the above equations, when the system is in stochastic equilibrium and state probabilities do not depend on time, is given in [44]

(6) $$f\left(x\right)=\left\{\begin{array}{ccc} \frac{\lambda p_{0}}{-\beta }\left(1-e^{zx}\right) & \;\;\;\;\mathrm{for}\;\;\;\;0<x\leq 1\;, & \\ \frac{\lambda p_{0}}{-\beta }\left(e^{-z}-1\right)e^{zx} & \;\;\;\;\mathrm{for}\;\;\;\;1\leq x\leq N-1\;, & \\ \frac{\mu p_{N}}{-\beta }\left(e^{z(x-N)}-1\right) & \;\;\;\;\mathrm{for}\;\;\;\;N-1\leq x<N\;, \end{array}\right.$$

where $z=\frac{2\beta }{\alpha }$. Normalization gives us probabilities $p_{0}$ and $p_{N}$.

The transient solution of Equations (3)–(5) may be obtained with an analytical-numerical algorithm proposed in [50], used and discussed, e.g., in [51] and recently in [48]. First, the diffusion equation is solved with absorbing barriers at x=0 and x=N; i.e., the process is ended when it reaches a barrier. The solution $\phi (x,t;x_{0})$ is [52]

(7) $$\phi \left(x,t;x_{0}\right)=\left\{\begin{array}{ccc} \delta (x-x_{0}) & \;\;\;\;\mathrm{for}\;\;\;\;t=0\;, & \\ \frac{1}{\sqrt{2\Pi \alpha t}}\sum_{n=-\infty }^{\infty }\left\{a\left(t\right)+b\left(t\right)\right\} & \;\;\;\;\mathrm{for}\;\;\;\;t>0\;, \end{array}\right.$$

where:

$$\begin{array}{c} a\left(t\right)=\exp\left[\frac{\beta x_{n}^{\prime }}{\alpha }-\frac{{\left(x-x_{0}-x_{n}^{\prime }-\beta t\right)}^{2}}{2\alpha t}\right],\\ b\left(t\right)=\exp\left[\frac{\beta x_{n}^{\prime \prime }}{\alpha }-\frac{{\left(x-x_{0}-x_{n}^{\prime \prime }-\beta t\right)}^{2}}{2\alpha t}\right], \end{array}$$

and $x_{n}^{\prime }=2nN$, $x_{n}^{\prime \prime }=-2x_{0}-x_{n}^{\prime }\;.$

Then, the density of the diffusion process having barriers with jumps is expressed with the use of functions $\phi (x,t;x_{0})$

(8) $$f\left(x,t;\psi \right)=\phi \left(x,t;\psi \right)+\int_{0}^{t}g_{1}\left(\tau \right)\phi \left(x,t-\tau ;1\right)d\tau +\int_{0}^{t}g_{N-1}\left(\tau \right)\phi \left(x,t-\tau ;N-1\right)d\tau \;.$$

where $g_{1}\left(t\right)$ and $g_{N}\left(t\right)$ are derived with the use of balance Equations (4) and (5).

This is the transient solution, but it assumes constant parameters of equations. If they are changing with time, e.g., if the flow intensity λ is time-dependent, and, in consequence, we have α(t) and β(t), the diffusion equation is solved in short time intervals where the parameters of the equation are considered constant and change their values only with the change in the interval. The solution at the end of an interval is used as the initial condition for the next interval.

The solution f(x,t) approximates the distribution of the queue length. The density of the queue latency (response time) is obtained with the use of the first passage time; i.e., the time the process needs to walk a certain distance. The density function $\gamma_{x_{0},0}\left(t\right)$ of the first passage time from $x=x_{0}$ to x=0,

(9) $$\begin{array}{ccc} \gamma_{x_{0},0}\left(t\right) & = & \frac{\partial }{\partial t}\int_{0+}^{\infty }\phi \left(s,t;x_{0}\right)dx=lim_{x\to 0}\left[\frac{\alpha }{2}\frac{\partial }{\partial x}\phi \left(x,t;x_{0}\right)-\beta \phi \left(x,t;x_{0}\right)\right]\\ & = & \frac{x_{0}}{\sqrt{2\Pi \alpha t^{3}}}e^{-\frac{{\left(x_{0}+\beta t\right)}^{2}}{2\alpha t}}. \end{array}$$

A new customer who joins the queue at time *t* has, with probability density f(x,t), *x* customers ahead him. The queueing delay is equivalent to the time the process needs to go from the initial point *x* to 0 (corresponding to the customer service). The pdf of the delay introduced by the queue length distribution with density f(ξ,t;ψ) is then

(10) $$f_{R}\left(x,t\right)=\int_{0}^{N}\gamma_{\xi ,0}\left(x\right)f\left(\xi ,t;\psi \right)d\xi .$$

The input traffic may be non-homogeneous, composed of independent flows called *classes*, k=0,1,…K that have input parameters $\lambda^{\left(k\right)}$, ${\sigma_{A}^{\left(k\right)}}^{2}$ specific to each class and service parameters $\mu^{\left(k\right)}$, ${\sigma_{B}^{\left(k\right)}}^{2}$ waiting for service in the common FIFO queue. In this case, the number of all class customers coming to the system has a normal distribution with mean and variance being the sum of corresponding means and variances. The input and service parameters for the total flow of customers are [53]

(11) $$\lambda =\sum_{k=0}^{L}\lambda^{\left(k\right)},\;C_{A}^{2}=\sum_{k=1}^{L}\frac{\lambda^{\left(k\right)}}{\lambda }{C_{A}^{\left(k\right)}}^{2}\;,\;\;\;\;\;$$

(12) $$\frac{1}{\mu }=\sum_{k=1}^{L}\frac{\lambda^{\left(k\right)}}{\lambda }\frac{1}{\mu^{\left(k\right)}}\;,\;C_{B}^{2}=\mu^{2}\sum_{k=1}^{L}\frac{\lambda^{\left(k\right)}}{\lambda }\;\frac{1}{{\mu^{\left(k\right)}}^{2}}\left({C_{B}^{\left(k\right)}}^{2}+1\right)-1\;,$$

where $\lambda^{\left(k\right)}/\lambda$ is the probability that a customer belongs to a class *k*. The diffusion process where α and β have the above parameters gives the approximation of p(n) and the distribution of the total number of customers in the station, and then for any class *k*, the distribution $p^{\left(k\right)}\left(\nu \right)$

(13) p(k)(ν)=∑n=νN[p(n)nν(λ(k)λ)ν(1−λ(k)λ)n−ν],k=0,…,L.

### 2.2. Preemptive-Resume G/G/1/N/PRIOR Station

The above classic model of G/G/1/N station with FIFO queue may be extended to the case of multiple classes of customers, with each class having its own priority. Depending on the type of priorities, the service of these clients is different. There are three categories of interrupted service queues: (1) postponable, (2) preemptive-resume, and (3) preemptive repeat. The first category assumes that when a new client with higher priority comes to the system, he waits for the end of the service of the currently serviced client (the current service is not interrupted). The second and third category assume interruption with the service of the currently serviced client and the start of the service of the new client. However, after the end of the service of the higher privileged clients, the preemptive-resume interruption continues the service of the client and preemptive-repeat starts the client service from the beginning. A good review of classical models of priority systems is given in [54]. It refers in general to M/G/1/PRIOR steady-state models. Below, we deal with preemptive-resume priorities. Our model, similarly to in the case of one class of customers, assumes general distributions of interarrival times and service times at each priority level and limited to *N* number of customers of each priority.

We keep the notation described in the previous section of adding upper index (k) to identify the priority class k=0,1,…L, k=0 as the highest priority, and k=L as the lowest. This way, $1/\lambda^{\left(k\right)}$ and $\sigma_{A}^{{\left(k\right)}^{2}}$ refer to the mean and variance of interarrival times of class *k* customers, and $1/\mu^{\left(k\right)}$ and $\sigma_{B}^{{\left(k\right)}^{2}}$ refer to the mean and variance of their service times; $p_{0}^{\left(k-1\right)}\left(t\right)$ is probability that at time *t* there are no customers of class *k* in the system.

We will also consider a diffusion process $X^{K}\left(t\right)$, which refers to the joint number of customers of classes 0…K in the system; parameters $\alpha^{K}$ and $\beta^{K}$ refer to its movement, and $f^{K}\left(x,t;x_{0}\right)$ denotes its pdf. With the same arguments as for one class in the previous section, we may say that the number of customers of several classes counted jointly at arrival and departure has a normal distribution. The diffusion process may describe the evolution of this number of customers in the system. However, only the input processes of these classes are independent. The output process of a class *k* is dependent on the processes of all higher classes: the service of a customer of class *k* may be finished only if customers of classes 0…k−1 are absent in the system. Therefore, the parameters $\alpha^{K}$, $\beta^{K}$ may be written as

(14) $$\begin{array}{ccc} \alpha^{K} & = & \sum_{k=1}^{K}\lambda^{\left(k\right)}C_{A}^{{\left(k\right)}^{2}}+\sum_{k=1}^{K}\left(\left(1-p_{0}^{\left(k-1\right)}\left(t\right)\right)\mu^{\left(k-1\right)}C_{B}^{{\left(k-1\right)}^{2}}\right)\\ & & +\sum_{k=1}^{K}\left(p_{0}^{\left(k-1\right)}\left(t\right)\mu^{\left(k\right)}C_{B}^{{\left(k\right)}^{2}}\right),\\ \beta^{K} & = & \sum_{k=1}^{K}\lambda^{\left(k\right)}-\sum_{k=1}^{K}\left(\left(1-p_{0}^{\left(k-1\right)}\left(t\right)\right)\mu^{\left(k-1\right)}\right)-\sum_{k=1}^{K}\left(p_{0}^{\left(k-1\right)}\left(t\right)\mu^{\left(k\right)}\right), \end{array}$$

where

$$\begin{array}{c} C_{A}^{{\left(k\right)}^{2}}=\sigma_{A}^{{\left(k\right)}^{2}}\lambda^{{\left(k\right)}^{2}},\;C_{B}^{{\left(k\right)}^{2}}=\sigma_{B}^{{\left(k\right)}^{2}}\mu^{{\left(k\right)}^{2}}. \end{array}$$

Let $v^{\left(K\right)}\left(n,t\right)$ denote the probability that *n* customers of class *K* are present at time *t* in the system, and let $p^{K-1}\left(n,t\right)$ denote the probability that *n* customers of all classes 0,…K−1 are present at time *t* in the system. Obviously, for the highest priority class

$$\begin{array}{c} v^{\left(0\right)}\left(n,t\right)=p^{0}\left(n,t\right) \end{array}$$

and for other classes

$$\begin{array}{c} p^{K}\left(n,t\right)=\sum_{\nu =0}^{n}p^{K-1}\left(n-\nu ,t\right)v^{\left(K\right)}\left(\nu ,t\right),\;K=1,\ldots ,L,\;n=0,1,\ldots ,N \end{array}$$

or

(15) $$v^{\left(K\right)}\left(n,t\right)=\frac{p^{K}\left(n\right)-\sum_{\nu =0}^{n-1}p^{K-1}\left(n-\nu ,t\right)v^{\left(K\right)}\left(\nu ,t\right)}{p^{K-1}\left(0,t\right)},\;K=1,\ldots ,L,\;n=0,1,\ldots ,N$$

Note that index *K* refers to classes 0,…,K and index (K) to the single class *K*.

Denote by $E[n^{\left(k\right)}\left(t\right)]$ the mean number of customers class *k* present in the system

$$\begin{array}{c} E\left[n^{\left(k\right)}\left(t\right)\right]=\sum_{\nu =0}^{\infty }v^{\left(k\right)}\left(\nu ,t\right)\nu \end{array}$$

and by $E[n^{K}\left(t\right)]$ the mean number of customers class 0…K in the system

$$\begin{array}{c} E\left[n^{K}\left(t\right)\right]=\sum_{n=0}^{\infty }p^{K}\left(n,t\right)n, \end{array}$$

A sketch of the algorithm is as follows:

- K=0: we consider the highest priority class k=0 alone and use the single class model presented in the previous section. The customers of lower classes are transparent for k=0 class; therefore, the solution is correct. $f^{\left(0\right)}\left(n,t;x_{0}\right)=p^{\left(0\right)}\left(n,t\right)=v^{\left(0\right)}\left(t\right)$.
- K=1: we consider two classes, k=0,1, determine $\alpha^{\left(1\right)}$, $\beta^{\left(1\right)}$ following (14), solve the diffusion equation with these parameters to obtain $f^{\left(1\right)}\left(x,t;x_{0}\right)$, which approximates the distribution $p^{1}\left(n,t\right)$ of the joint number of customers of classes k=0 and k=1; we then compute $v^{\left(1\right)}\left(n,t\right)$.
- K=2: we consider the system with three classes, k=0,1,2 to determine the parameters $\alpha^{\left(2\right)}$, $\beta^{\left(2\right)}$ following (14), solve the diffusion equation to obtain $f^{2}\left(x,t;x_{0}\right)$ and $p^{2}\left(n,t\right)$, then, using $p^{\left(1\right)}\left(n,t\right)$ of the previous step, compute $v^{\left(2\right)}\left(n,t\right)$, etc., until K=L.

Note that for the mean values, $E\left[n^{K}\left(t\right)\right]=E\left[n^{K-1}\left(t\right)\right]+E\left[n^{\left(K\right)}\left(t\right)\right]$.

Before analyzing the waiting times, we have to define the distribution of the completion time. The completion time is the period between the start and the end of any customer service. On the highest priority level, the completion time is equal to the service time; for other classes, it also includes interruptions caused by the arrival and service of higher-priority customers. Suppose *T* is the service time of a customer of class *k*. If *n* customers of classes 1,…,k−1 arrive during the time *T*, the service will be interrupted *n* times; *n* has an approximately normal distribution with the mean $\sum_{l=0}^{k-1}\lambda^{\left(l\right)}T$ and the variance $\sum_{l=0}^{k-1}\lambda^{\left(l\right)}C_{A}^{{\left(l\right)}^{2}}T$.

The duration of any of *n* breaks is distributed like the busy period $\gamma^{\left(k-1\right)}$ of the system serving customers of classes 0,…,k−1. The busy period starts with the arrival of a customer to the empty system and lasts until the moment when the system becomes empty. Its duration may be seen as the first passage time from $x_{0}=1$ (first customer arrives) to x=0 (nobody in the system) and is given by Equation (9) with parameters corresponding to the diffusion process with K−1 classes. For the sake of simplicity, we neglect here the weak probability that the process, before it comes to zero, may reach the upper barrier at *N*, stay there, jump to N−1, come back to *N*, etc.

The total time of breaks in *T* has the pdf

$$\begin{array}{c} \varphi^{\left(k\right)}\left(t|T\right)=\sum_{n=0}^{\infty }p_{n|T}\gamma^{\left(k-1)(*n\right)}\left(x\right) \end{array}$$

where $p_{n|T}$ is the probability of *n* breaks in *T* and $\gamma^{\left(k-1)(*n\right)}\left(t\right)$ is the *n*-fold convolution of $\gamma^{\left(k-1\right)}\left(t\right)$ with itself. Thus the pdf $c^{\left(k\right)}\left(t\right)$ of the completion time is

$$\begin{array}{c} c^{\left(k\right)}\left(t\right)=\int_{0}^{\infty }b^{\left(k\right)}\left(t\right)\varphi^{\left(k\right)}\left(t-T|T\right)1\left(t-T\right)dT, \end{array}$$

where 1(t)=0 for t<0 and 1(t)=1 for t≥0, and from its Laplace transform

$$\begin{array}{c} c^{\left(k\right)}\left(s\right)=\int_{0}^{\infty }b^{\left(k\right)}\left(T\right)e^{-sT}\sum_{n=0}^{\infty }\left\{p_{n/T}{\left[{\bar{\gamma }}^{\left(k\right)}\left(s\right)\right]}^{n}\right\}dT \end{array}$$

we obtain its moments $E=[c^{\left(k\right)}]$ and $E[{\left(c^{\left(k\right)}\right)}^{2}]$:

$$\begin{array}{ccc} E=[c^{\left(k\right)}] & = & -\frac{d}{ds}c^{\left(k\right)}{\left(s\right)}_{s=0}=\left\{E\left[\gamma^{\left(k-1\right)}\right]\Lambda^{\left(k-1\right)}+1\right\}\frac{1}{\mu^{\left(k\right)}},\\ E[{\left(c^{\left(k\right)}\right)}^{2}] & = & \frac{d^{2}}{ds^{2}}c^{\left(k\right)}{\left(s\right)}_{s=0}=E{\left[\gamma^{\left(k-1\right)}\right]}^{2}[\left(\sum_{l=0}^{\left(k-1\right)}\lambda^{\left(l\right)}C_{A}^{{\left(l\right)}^{2}}\right)\frac{1}{\mu^{\left(k\right)}}\\ & & -\Lambda^{\left(k-1\right)}\frac{1}{\mu^{\left(k\right)}}]+E\left[{\left(\gamma^{\left(k-1\right)}\right)}^{2}\right]\Lambda^{\left(k-1\right)}\frac{1}{\mu^{\left(k\right)}}+\\ & + & E\left[\gamma^{\left(k-1\right)}\right]E\left[{\left(b^{\left(k\right)}\right)}^{2}\right]\Lambda^{\left(k\right)}\cdot \\ & & \cdot \left\{E\left[\gamma^{\left(k-1\right)}\right]\Lambda^{\left(k-1\right)}+2\right\}+E\left[{\left(b^{\left(k\right)}\right)}^{2}\right]. \end{array}$$

where $\Lambda^{\left(k\right)}=\sum_{l=0}^{k}\lambda^{\left(l\right)}$.

When all input streams are Poisson, i.e., $C_{A}^{{\left(l\right)}^{2}}=1$, l=1,…,k, the results are identical to the exact formulae given for this case in [54].

Finally, similarly as in Equation (10), we can define the pdf of the delay (response time) at every priority level *k*:

(16) $$f_{R^{\left(k\right)}}\left(x,t\right)=\int_{0}^{N}\gamma_{\xi ,0}^{\left(k\right)}\left(x\right)f^{\left(k\right)}\left(\xi ,t;\psi \right)d\xi .$$

In the pdf of the first passage time $\gamma_{\xi ,0}^{\left(k\right)}\left(x\right)$ for a priority *k*, the mean and variance of the service time should be replaced by the mean and variance of the completion time $c^{\left(k\right)}$. Mean waiting time $E[w^{\left(k\right)}]$ to start the service is

$$\begin{array}{c} E\left[w^{\left(k\right)}\right]=E\left[R^{\left(k\right)}\right]-E\left[c^{\left(k\right)}\right]. \end{array}$$

## 3. Validation of the Priority Model

Diffusion approximation remains a heuristic approach, and we do not know strict bounds on its errors; therefore, we should check its quality in various cases. The errors of the method in case of FIFO queue as presented in Section 2.1 were discussed, e.g., in [46,51]. Below, we investigate the quality of the diffusion priority model, considering a few numerical examples differing in the number of priorities, input intensities, and type of interarrival and service time distributions.

The first three cases concentrate on various input intensities and two priority classes. The fourth case investigates a system with three priorities. The fifth and final scenario is dedicated to non-exponential interarrival distribution. In all cases, the results of the diffusion model have been validated by comparison with the ones obtained with OMNeT++ discrete network simulator [55]. The standard OMNET++ package can only collect steady-state results; therefore, we adapted it to the needs of transient analysis by modifying packet generators, algorithms of collecting statistics, and handling message mechanisms. The simulation results are averaged over 100,000 independent runs.

### 3.1. Two Priorities, Low Input Intensities

This scenario considers two classes of customers. The arrival rate of the priority class changes in the following way: $\lambda^{\left(0\right)}=0.4$ during intervals t∈[0,10],[20,30],[40,50],…, and $\lambda^{\left(0\right)}=0$ between these intervals. Time is expressed in generic units. The non-priority customers arrive with constant intensity $\lambda^{\left(1\right)}=0.4$. Both queues are limited to $N^{\left(0\right)}=N^{\left(1\right)}=20$; i.e., the system can host up to 20 customers of each class but no more than 20 in total. When active, the input streams are Poisson; exponential service time distributions for both classes are the same: $\mu^{\left(0\right)}=\mu^{\left(1\right)}=1$. The system is stable because its maximum utilization factor is ρ=0.8, but the considered intervals are too short to allow it to attain a steady-state.

**Figure 1:**
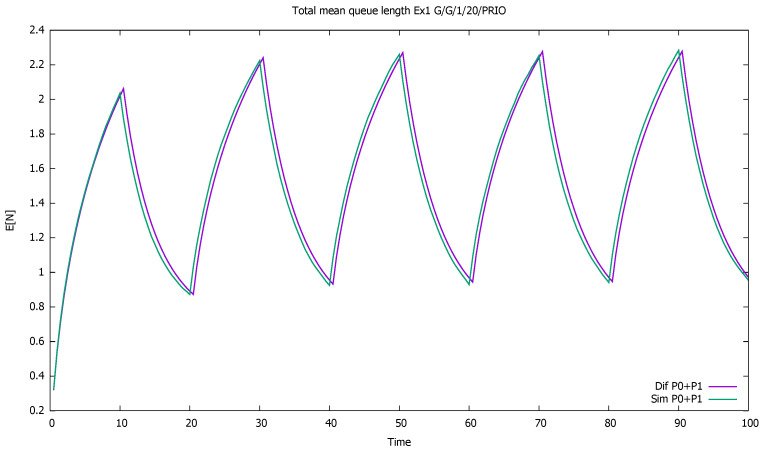
Section 3.1, low load: total mean queue length as a function of time for both classes (P0 + P1) taken together.

The total number of customers of both classes displayed in Figure 1 increases when the priority customers come into the system. In the remaining periods of time, the service (μ=1) is fast enough to countermeasure the non-priority intensity (λ=0.4) and makes the queue effectively decrease until new priority clients arrive at the system.

**Figure 2:**
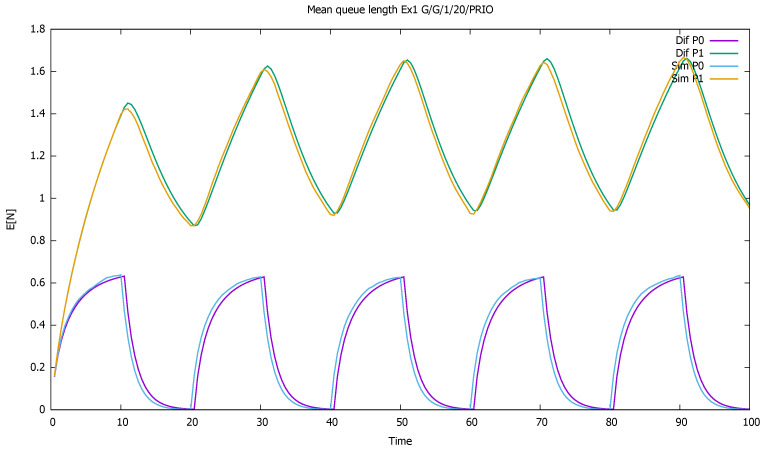
Section 3.1, low load: mean queue lengths as a function of time for priority (P0) and non-priority (P1) classes, and diffusion and simulation results.

Figure 2 displays the mean number of customers of each class as a function of time. The diffusion and simulation results are compared. We see how when both flows are active, mainly priority customers are served, and the non-priority clients are queued and wait their turn—their queue increases almost linearly. For every 10 time units in which priority customers do not come, they have more chance to enter the service, and their queue empties.

**Figure 3:**
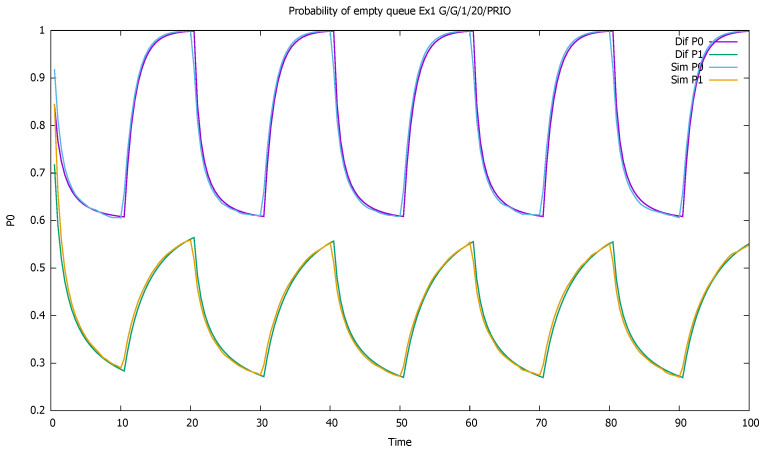
Section 3.1, low load: probabilities of empty queues for priority (P0) and non-priority (P1) classes.

Figure 3 shows probabilities $p_{0}^{\left(0\right)}\left(t\right)$ and $p_{0}^{\left(1\right)}\left(t\right)$ of empty queue for both classes. The probabilities decrease during higher traffic periods and increase elsewhere; we see the influence of the priority traffic on non-priority traffic, with a constant arrival rate.

**Figure 4:**
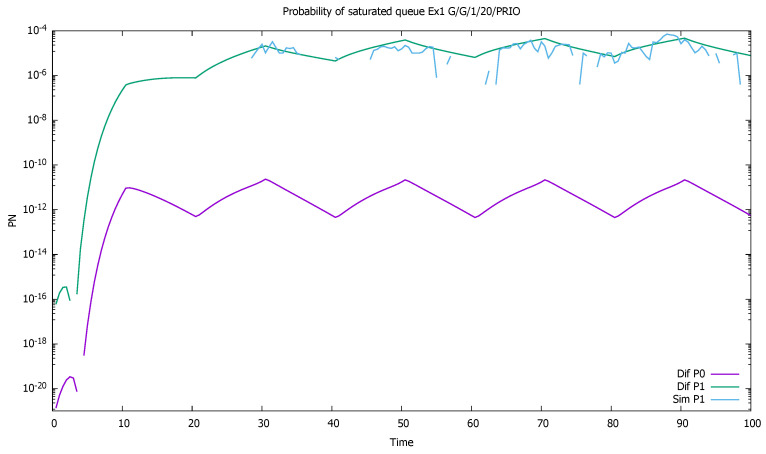
Section 3.1, low load: probabilities of saturated queues as a function of time for priority (P0) and non-priority (P1) classes.

Similarly, probabilities $p_{N}^{\left(0\right)}\left(t\right)$ and $p_{N}^{\left(1\right)}\left(t\right)$ are displayed in Figure 4. As the probability of buffer overflow is weak, we observe that the series of 100,000 simulation runs is not sufficient to determine it properly. No overflow is observed in simulation for the priority class, and the simulation results for the non-priority class are distorted. There is no numerical problems in case of diffusion approximation, even if results are in the order of $10^{-18}$.

### 3.2. Two Priorities, Medium Input Intensities

We keep the same pattern of the traffic but increase its intensity. The rate of priority traffic is ($\lambda^{\left(0\right)}=1.2$), i.e., three times higher than previously in Section 3.1 in the same intervals t∈[0,20],[40,60],[80,100] and zero otherwise. The intensity of the non-privileged class is higher by 0.1 ($\lambda^{\left(1\right)}=0.5$), and constant.

**Figure 5:**
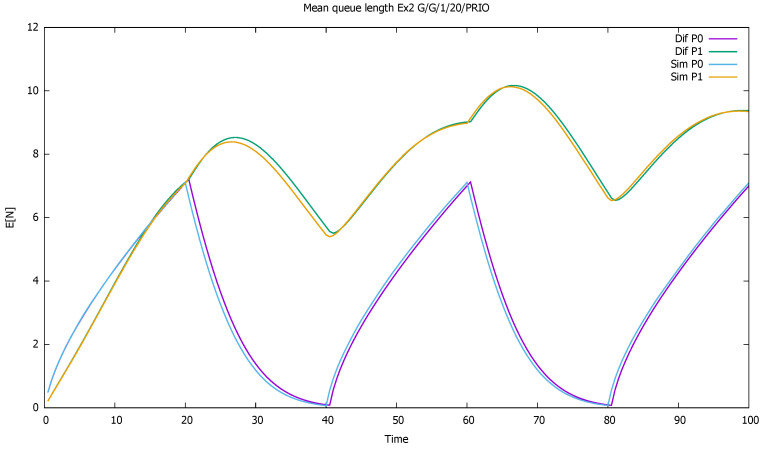
Section 3.2, medium load: mean queue lengths of priority (P0) and non-priority (P1) classes.

**Figure 6:**
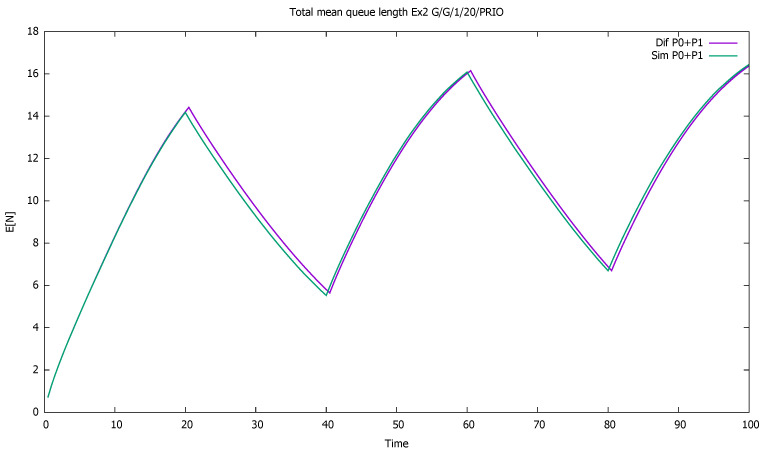
Section 3.2, medium load: total mean queue length for both classes together.

The system is unstable during active periods of priority traffic; the service station is not able to serve all incoming priority customers. During every first 20 time-units in 40-unit cycles, the first-class customers are serviced and queued, unlike second-class customers, who are only queued. For the next seven time units, on average, the class 1 service continues, which results in further queuing of class 2. During the last 13 units of the cycle, on average, the service of priority clients ends, and the non-priority begins. However, the low traffic period is too short to allow the service of all accumulated non-priority customers. The lower priority queue is gradually increasing cycle by cycle, and the same is true for the total number of customers of both classes; see Figure 5 and Figure 6.

**Figure 7:**
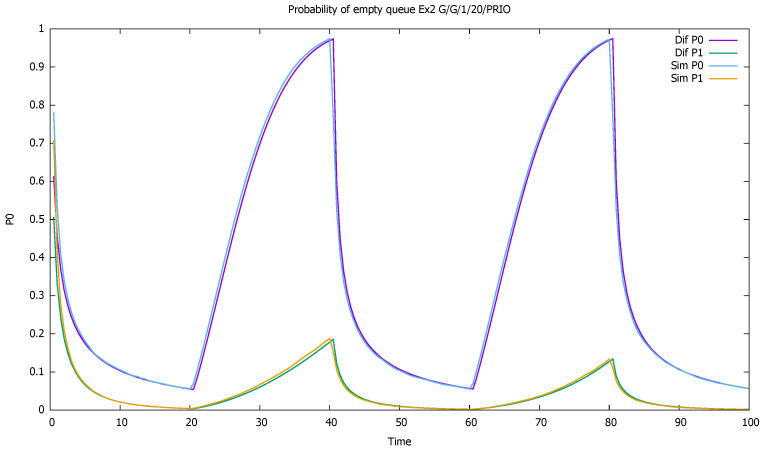
Section 3.2, medium load: probabilities of empty queue $p_{0}\left(t\right)$ for priority (P0) and non-priority (P1) classes.

**Figure 8:**
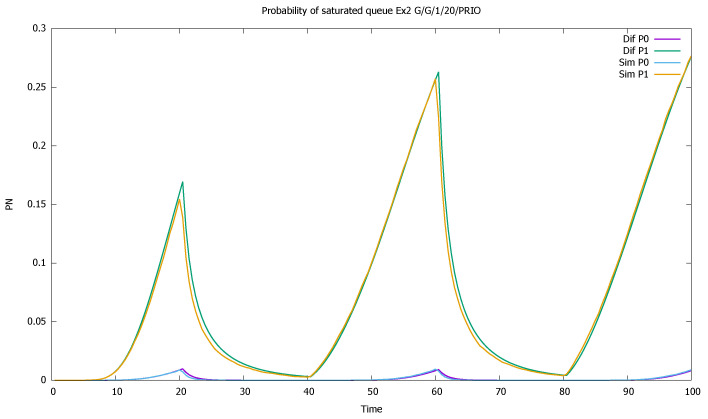
Section 3.2, medium load: probabilities of saturated queues $p_{N}\left(t\right)$ for priority (P0) and non-priority (P1) classes.

The changes in the system highly affect the probability of both empty (Figure 7) and full (Figure 8) queues of classes 1 and 2. Again, the observation starts with an empty queue, but this time the probability of an empty queue for non-priority drops to zero. Moreover, the probability that the saturated non-priority queue increases with each cycle is grows a little bigger, signaling that the system will saturate in the future.

### 3.3. Two Priorities, High Input Intensities

The input intensity is two times higher ($\lambda^{\left(0\right)}=2.4$) than in the previous Section 3.2. The lower class intensity remains constant and is twice as high previously, ($\lambda^{\left(1\right)}=1$). The intervals and other assumptions remain the same as in Section 3.2.

**Figure 9:**
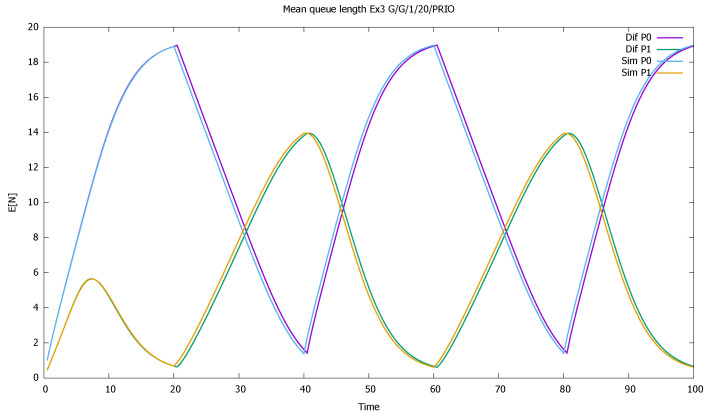
Section 3.3, high load: mean queue lengths of priority (P0) and non-priority (P1) classes.

**Figure 10:**
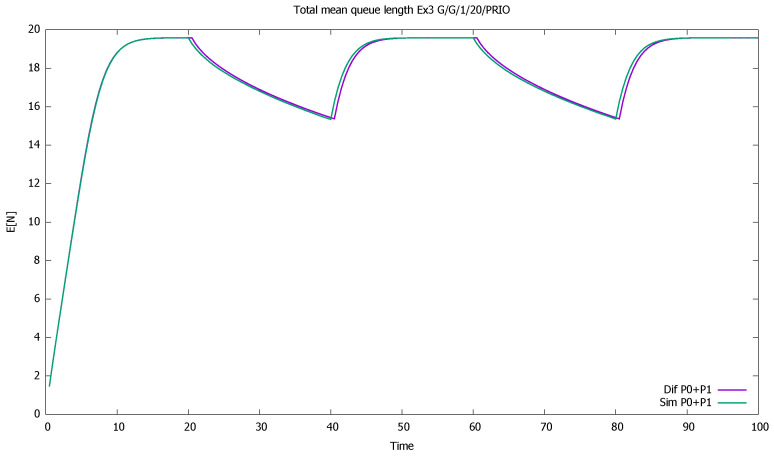
Section 3.3, high load: total mean queue length of both priority classes.

**Figure 11:**
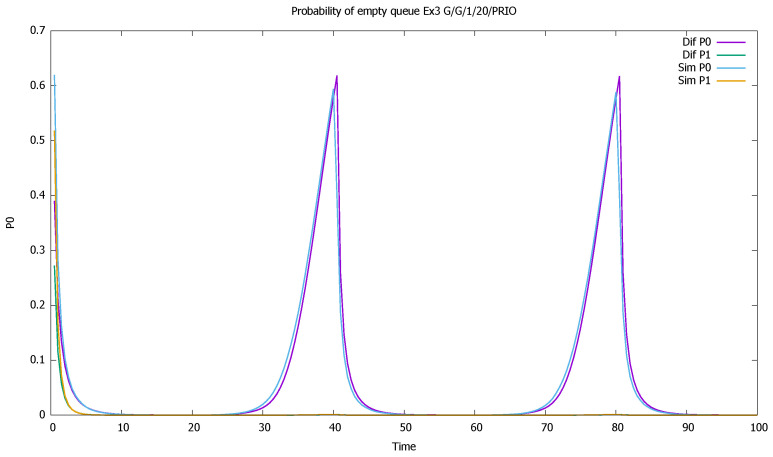
Section 3.3, high load: probabilities of empty queues for priority (P0) and non-priority (P1) classes.

**Figure 12:**
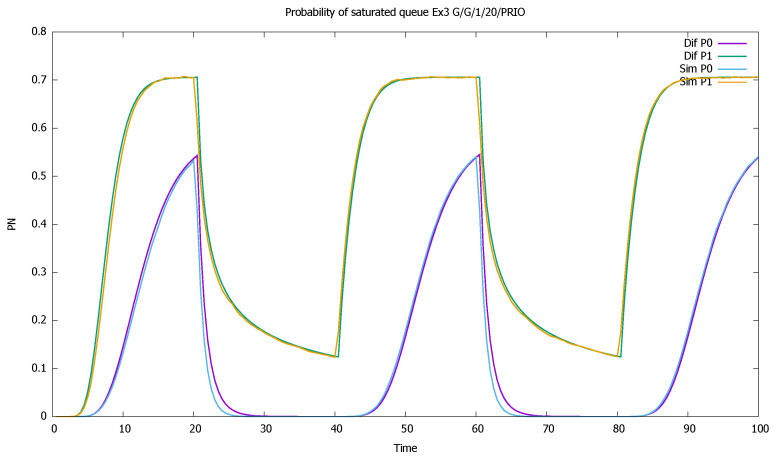
Section 3.3, high load: probabilities of saturated queues for priority (P0) and non-priority (P1) classes.

Initially, both queues increase, as shown in Figure 9. After a while, the non-priority queue drops almost to zero because the buffer is monopolized by the higher class. Only when priority customers cease to arrive do lower-priority clients have a chance to enter the buffer, and their queue increases. Therefor the cycles of queue changes are interleaved: when the priority queue increases, the non-priority queue decreases, and vice-versa. There is not space enough in the buffer for both classes; Figure 10 shows that for most of the time, it is full or almost full. The system remains stable due to massive losses; see probabilities of the empty queue in Figure 11 and Figure 12. The probability that the priority queue is full reaches its maximum at the ends of the customer arrival cycles and then decreases. Only at these moments do the lower-class clients see that the queue may be available.

### 3.4. Three Priorities; Mean Service Time Depends on Priority 

This time, the server is designed to handle three priority levels. The highest-priority customers come with intensity $\lambda^{\left(0\right)}=0.25$ during the same intervals as in Section 3.2 and Section 3.3 and otherwise $\lambda^{\left(0\right)}=0$. The intensity of medium-priority customers is constant, $\lambda^{\left(1\right)}=0.5$, while the lowest-priority customers have constant intensity $\lambda^{\left(3\right)}=0.25$. The service rates are $\mu^{\left(0\right)}=\mu^{\left(2\right)}=1$ and $\mu^{\left(1\right)}=0.5$. Queue capacities are limited to $N^{\left(0\right)}=N^{\left(1\right)}=N^{\left(3\right)}=20$. This means that the system as a whole is unstable.

**Figure 13:**
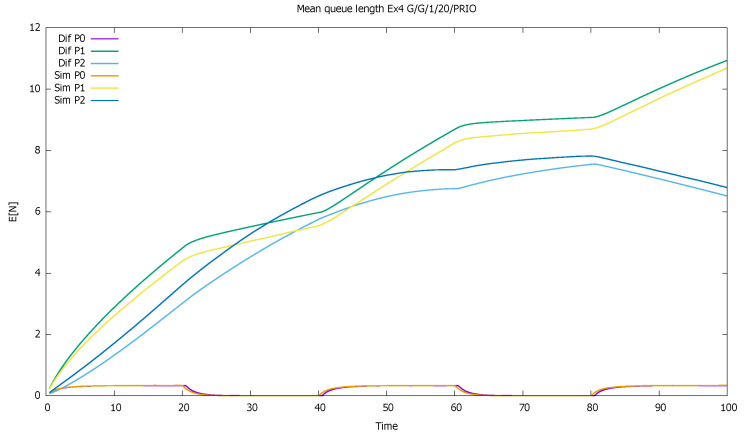
Section 3.4, three priorities: mean queue lengths as a function of time for priority (P0), medium-priority (P1), and low-priority classes.

The highest-priority class (class 0) has a small queue because the station is four times faster than the rate of its arrivals. For class 1, the utilization equals one; i.e., the medium priority queue will slowly increase up to the buffer limit. The lowest-priority class must wait for the first and second class to free the space; at the beginning, its mean queue increases, but then this class is gradually eliminated from service as the medium class fills the buffer. The process is presented in Figure 13.

**Figure 14:**
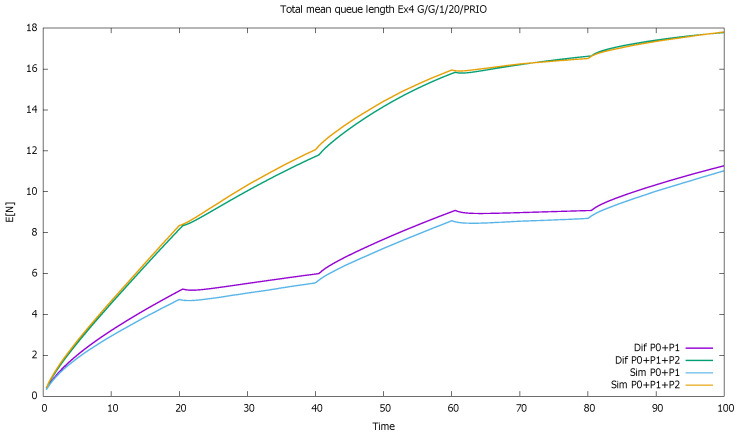
Section 3.4, three priorities: total mean queue length as a function of time of three priority classes.

Figure 14 compares the total number of customers of classes in two groups: (1) high and medium priority and (2) all three priorities together. Both groups contain medium class, which constantly increases and it results in the increase in both total queues.

**Figure 15:**
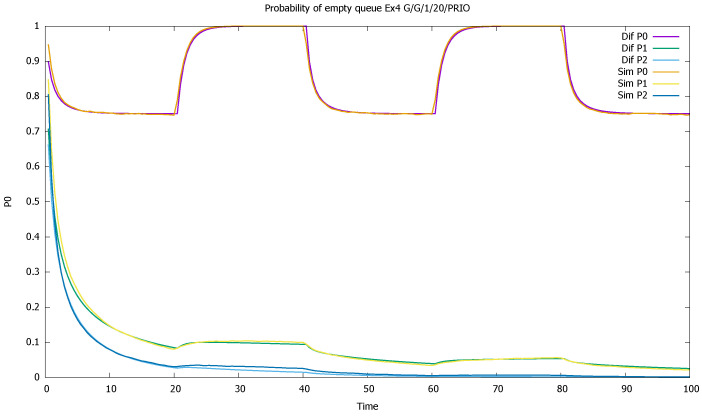
Section 3.4, three priorities; probabilities of empty queues as a function of time for three priority classes (P0), (P1), (P2).

The probability of the empty queue is close to one for the highest-priority class and changes periodically with the active and non-active traffic intervals of this class. For the other classes, this probability is constantly decreasing, as seen in Figure 15.

**Figure 16:**
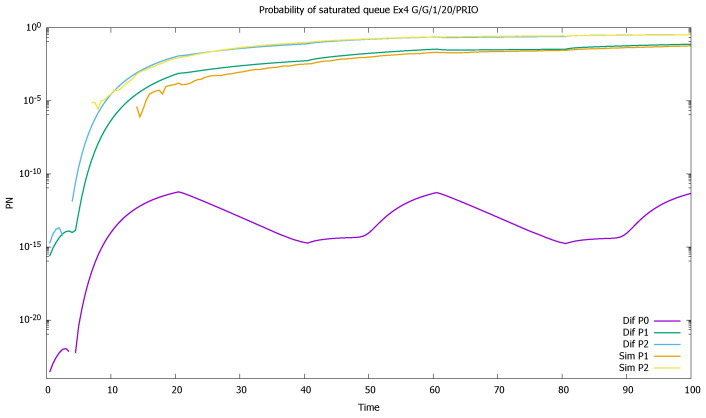
Section 3.4, three priorities: probabilities of saturated queues as a function of time; in case of P(0) only diffusion results are available, the simulations were too short to give such small values.

The probability of a full queue is so small for the highest class (P0) that we did not receive it in simulations; see Figure 16. For medium and low priorities, this probability increases with time and is much faster in the case of P(2).

### 3.5. Two Priorities; General Interarrival and Service Time Distributions 

This example considers a server with two priority levels; the input traffic is non-Poisson for both classes. The priority customers come with intensity $\lambda^{\left(0\right)}=0.75$, and the squared coefficient of variation of their interarrival time distribution is $C_{A}^{{\left(0\right)}^{2}}=8$ during intervals t∈[0,20],[40,60],[80,100]. Otherwise, $\lambda^{\left(0\right)}=0$. The intensity of non-priority traffic is constant, $\lambda^{\left(1\right)}=0.75$, with squared coefficient of variation $C_{A}^{{\left(1\right)}^{2}}=5$. The queue capacities are limited to $N^{\left(0\right)}=N^{\left(1\right)}=20$.

**Figure 17:**
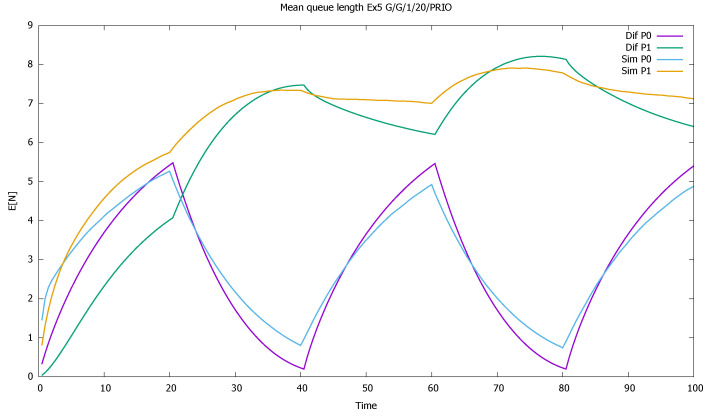
Section 3.5: mean queue lengths of priority (P0) and non-priority (P1) classes for very high values of $C_{A}^{2}$, $C_{B}^{2}$.

**Figure 18:**
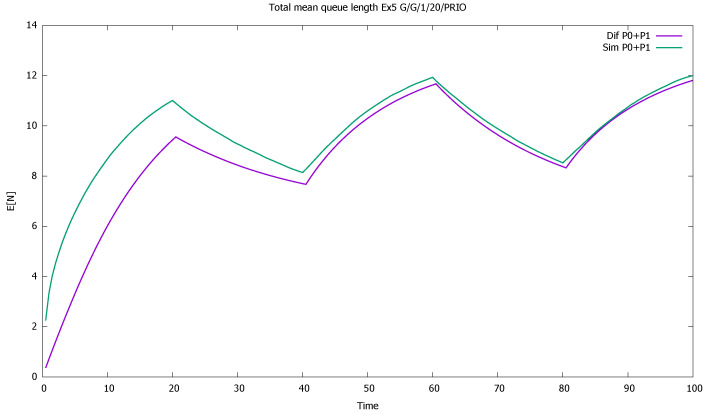
Section 3.5: total mean queue length of both priority classes for very high values of $C_{A}^{2}$, $C_{B}^{2}$.

**Figure 19:**
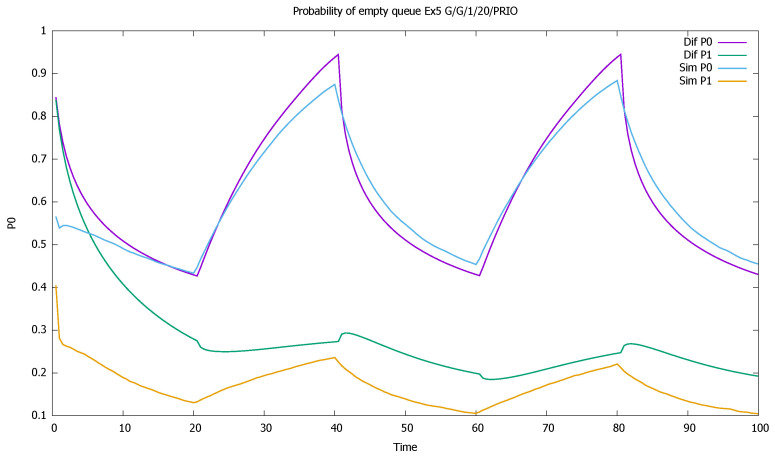
Section 3.5: probabilities of empty queues for priority (P0) and non-priority (P1) classes for very high values of $C_{A}^{2}$, $C_{B}^{2}$.

**Figure 20:**
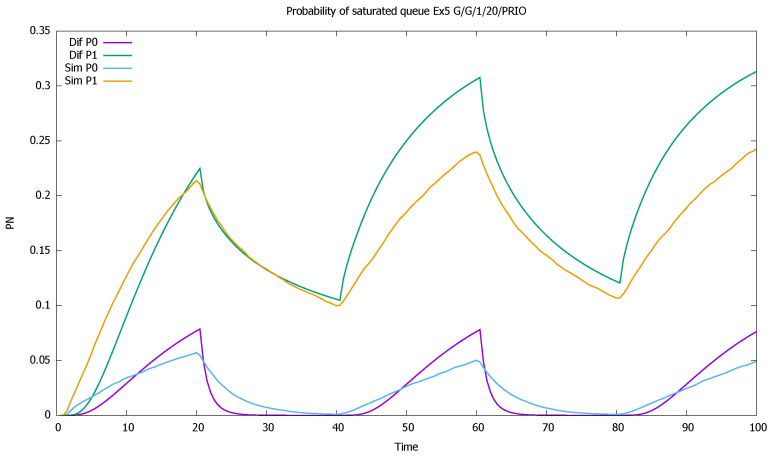
Section 3.5: probabilities of saturated queues for priority (P0) and non-priority (P1) classes for very high values of $C_{A}^{2}$, $C_{B}^{2}$.

The service rates are $\mu^{\left(0\right)}=\mu^{\left(1\right)}=1$, and the distributions of service time have $C_{B}^{{\left(0\right)}^{2}}=8$, $C_{B}^{{\left(1\right)}^{2}}=5$. Such high values of $C_{A}^{2}$, $C_{B}^{2}$ are not to be observed in real traffic: they are usually below 2. It is known that the errors of the approximation increase with the value of $C_{A}^{2}$, $C_{B}^{2}$ [46,56]; therefore we wanted to check the accuracy of the model for an extreme set of parameters. In diffusion approximation, the type of distributions is not important, only the value of its first two moments. In simulation we used Cox distributions with the same moments. For another distribution, the simulation results would be slightly different. As in previous examples, we present mean queue lengths of priority and non-priority customers (Figure 17), mean queue length of both priority classes together (Figure 18), probabilities of empty queues for priority and non-priority classes (Figure 19), and probabilities of saturated queues for priority and non-priority classes (Figure 20).

The results confirm the deterioration of the approximation: diffusion results are not as close to simulations as was the case previously, when we assumed that $C_{A}^{2}=C_{B}^{2}=1$; the impact of the squared coefficients of variation is visible. Note also that all model computations are performed inside small time intervals of one unit length, and the approximate distribution of the queue length at the end of one interval gives approximated initial conditions for the next one, increasing the deficiencies of the model. However, the results are still useful in the evaluation of a time-dependent behavior of the system and follow the general pattern given by simulation. A better match is observed for the priority class. It is also natural, as the evolution of the non-priority queue is based on the previous estimation of priority queue, and the errors add up.

The system is slightly unstable; therefore, mean queues slowly increase from one cycle to another (Figure 18). In addition, probabilities of queue saturation increase, as shown in Figure 19, and probabilities of the empty queue decrease with time, as shown in Figure 20.

## 4. Network of Priority and Non-Priority Queues

The steady-state diffusion model of an open network of G/G/1 or G/G/1/N queues was presented in [53], and it was adapted to transient states in, e.g., [57] and time-dependent routing in [48]. It is here extended to a time-dependent model of a network including both FIFO and priority stations. The approach is based on the decomposition of the network: we need to determine the input flow parameters at each station; then, we may use models of separate stations as discussed in Section 2.

Let *M* be the number of stations and L+1 the number of classes, k=0,1,…L. The traffic intensity $\lambda_{i}^{\left(k\right)}$ of class *k* at station *i* is determined by the system of M(L+1) equations representing the balance of flows:

(17) $$\lambda_{i}^{\left(k\right)}=\lambda_{0i}^{\left(k\right)}+\sum_{j=1}^{M}\sum_{l=1}^{l=L}\lambda_{j}^{\left(l\right)}r_{ji}^{lk},\;i=1,\ldots ,M,\;l=0,\ldots ,L.$$

where $r_{ji}^{lk}$ is the probability that a customer who belongs to station *j* and to class *l* goes next to the station *i* as a class *k* customer and $\lambda_{0i}^{\left(k\right)}$ is an external flow coming to station *i*.

To obtain the variance of interarrival times at any station *i*, we have to express it by the variances of interdeparture times, i.e., $C_{Dj}^{2}$ or ${C_{Dj}^{\left(l\right)}}^{2}$ at all stations sending customers to station *i*. In addition, we need to express the variance of interdeparture times at any station by the variance of interarrival times at the same station.

Both dependencies mutually relating the input and output of the stations turn out to be linear with respect to $C_{Dj}^{2}$ and $C_{Ai}^{2}$, and the simultaneous solution of the resulting system of equations brings us $C_{Ai}^{2}$ or ${C_{Ai}^{\left(k\right)}}^{2}$.

In transient analysis of the whole network, these equations are to be solved in time intervals that are sufficiently short to consider the flows, routing probabilities, and station utilization as constant parameters. We also distinguish input and output flows of a station; the output is changing continuously with changes in utilization $\varrho_{i}\left(t\right)$.

Assuming that the arrivals to a station *i* from other stations and from outside the network are independent, and assuming the variances of the arrivals from all directions, we come to the expression (18); see, e.g., Reference [48] for details. The variance of interarrival times at each station is obtained due the equations defining the variance of interdeparture times as a function of the parameters of the interarrival times at each station:

(18) $${C_{Aj}^{\left(l\right)}}^{2}=\frac{1}{\lambda_{j}^{\left(l\right)}}\sum_{i=1}^{M}\sum_{k=1}^{L}r_{ij}^{kl}\lambda_{i}^{\left(k\right)}\left[\left({C_{Di}^{\left(k\right)}}^{2}-1\right)r_{ij}^{\left(kl\right)}+1\right]+\frac{{C_{0j}^{\left(l\right)}}^{2}\lambda_{0j}^{\left(l\right)}}{\lambda_{j}^{\left(l\right)}}\;,$$

where ${C_{0j}^{\left(l\right)}}^{2}$ and $\lambda_{0j}^{\left(l\right)}$ refer to the flows coming from outside the network to station *j* as the first station, or for all classes together:

(19) $$C_{Aj}^{2}=\frac{1}{\lambda_{j}}\sum_{l=1}^{L}\lambda_{j}^{\left(l\right)}{C_{Aj}^{\left(l\right)}}^{2}.$$

The second type of equations linking the variances of $f_{Aj}\left(x\right)$ and $f_{Dj}\left(x\right)$, where $f_{Dj}\left(x\right)$ is the pdf of interdepature times at station *j*, will be discussed separately for FIFO and priority stations.

### 4.1. The Output Stream at the FIFO Station

The equations are based on Burke theorem [58]: if a station is active (i.e., it occurs with probability ϱ), the customers leave it in intervals equal to service times; otherwise we should wait for somebody to come and then serve them:

(20) $$f_{Dj}\left(x\right)=\varrho_{j}f_{Bj}\left(x\right)+\left[1-\varrho_{j}\right]f_{Aj}\left(x\right)*f_{Bj}\left(x\right)\;,\;j=1,\ldots ,M,$$

where $f_{Aj}\left(x\right)$ and $f_{Bj}\left(x\right)$ are density functions of interarrival and service times distributions at station *j* and * is the convolution. If the input flow is not Poisson, the use of interarrival time density $f_{Aj}\left(x\right)$ is an approximation; in fact it should be the pdf of idle time distribution. From Equation (20), we obtain

(21) $$C_{Dj}^{2}=\varrho_{j}^{2}\left(t\right)C_{Bj}^{2}+C_{Aj}^{2}\left(1-\varrho_{j}\right)+\varrho_{j}\left[1-\varrho_{j}\right]\;.$$

and

(22) $${C_{Dj}^{\left(k\right)}}^{2}=\frac{\lambda_{j}^{\left(k\right)}}{\lambda_{j}}\left(C_{Dj}^{2}-1\right)+1\;;$$

### 4.2. The Output Stream at the Priority Station

To use the same as the above schema in the case of priority stations, we need to develop an expression corresponding to Equation (21)—the distribution of interarrival times at each priority level. To simplify the notation, we omit here the index *i* denoting the station. Let us denote $f_{D}^{\left(k\right)}\left(x\right)$ as the pdf of interdeparture times in the stream of class *k* customers. It can be expressed as

(23) $$\begin{array}{ccc} f_{D}^{\left(k\right)}\left(x\right) & = & \frac{\varrho^{\left(k\right)}}{1-R^{\left(k-1\right)}}c^{\left(k\right)}\left(x\right)+\left(1-\frac{\varrho^{\left(k\right)}}{1-R^{\left(k-1\right)}}\right)\\ & & \times [\left(1-R^{(k-)1}\right)f_{A}^{\left(k\right)}\left(x\right)*c^{\left(k\right)}\left(x\right)\\ & & +R^{\left(k-1\right)}f_{A}\left(k\right)\left(x\right)*\gamma^{\left(k-1\right)}\left(t\right)*c^{\left(k\right)}\left(x\right)], \end{array}$$

where $R^{\left(k\right)}=\sum_{l=0}^{l=k}\varrho^{\left(l\right)}$, $\varrho^{\left(l\right)}=\lambda^{\left(l\right)}/\mu^{\left(l\right)}$.

The components of this expression correspond to three situations that are possible after the departure of any customer of class *k*:

– The next customer in the class *k* is in the system (this occurs with probability $\frac{\varrho^{\left(k\right)}}{1-R^{\left(k-1\right)}}$) and will leave it after its completion time;
– There are no customers of this class in the system, and we shall wait for the time described by $F_{A}^{\left(k\right)}\left(x\right)$ until it appears and enters the server;
– No customer of class *k* is present in the system, and a customer of higher class comes before him, so the busy period $\gamma^{\left(k-1\right)}$ must first be terminated.

From the above (23), we calculate the squared coefficient of the variation of interdeparture times for each priority customer, which is needed to integrate a single priority station into a network of such stations. The easiest way to obtain the moments of $f_{D}^{\left(k\right)}\left(x\right)$ given by Equation (23) is to use its Laplace transform ${\bar{f}}_{D}^{\left(k\right)}\left(s\right)$ and a formula that is valid for any density function $f_{X}\left(t\right)$ and its Laplace transform ${\bar{f}}_{X}\left(s\right)$

$$\frac{d^{n}{\bar{f}}_{X}^{\left(s\right)}}{ds^{n}}{|}_{s=0}=-\frac{d^{n}}{ds^{n}}\int_{0}^{\infty }f_{X}\left(x\right)e^{-sx}dx=\int_{0}^{\infty }f_{X}\left(x\right){\left(-1\right)}^{n}x^{n}e^{-sx}dx={\left(-1\right)}^{n}E\left[X^{n}\right]\;.$$

The final formula is as follows:

(24) $$\begin{array}{c} {C_{D}^{\left(k\right)}}^{2}=\sum_{l=1}^{k}h^{\left(k,l\right)}{C_{A}^{\left(l\right)}}^{2}+\psi^{\left(k\right)} \end{array}$$

where

$$\begin{array}{ccc} h^{\left(k,l\right)}\;\; & = & \;\;\left\{\begin{array}{cc} \left(\zeta^{\left(k,l\right)}+\frac{1-R^{\left(k\right)}}{1-R^{\left(k-1\right)}}R^{\left(k-1\right)}g^{\left(k-1,l\right)}\right){\left(\lambda^{\left(k\right)}\right)}^{2}, & l<k,\\ \frac{1-R^{\left(k\right)}}{1-R^{\left(k-1\right)}}, & l=k, \end{array}\right. \end{array}$$

and

$$\begin{array}{ccc} \zeta^{\left(k,l\right)} & = & \frac{\lambda^{\left(l\right)}}{\mu^{\left(k\right)}{\left(\beta^{\left(k-1\right)}\right)}^{2}}+g^{\left(k-1,l\right)}\frac{\Lambda^{\left(k-1\right)}}{\mu^{\left(k\right)}},\\ g^{\left(k,l\right)} & = & \frac{1}{{\left(\beta^{\left(k\right)}\right)}^{3}}, \end{array}$$

$$\begin{array}{ccc} \psi^{\left(k\right)} & = & \chi^{\left(k\right)}{\left(\lambda^{\left(k\right)}\right)}^{2}+\frac{1-R^{\left(k\right)}}{1-R^{\left(k-1\right)}}\{1+R^{\left(k-1\right)}e^{\left(k-1\right)}{\left(\lambda^{\left(k\right)}\right)}^{2}\\ & & +2\varrho^{\left(k\right)}\left(1-\frac{\Lambda^{\left(k-1\right)}}{\beta^{\left(k-1\right)}}\right)+\\ & & -\frac{\lambda^{\left(k\right)}R^{\left(k-1\right)}}{\beta^{\left(k-1\right)}}\left[1+2\varrho^{\left(k\right)}\left(1-\frac{\Lambda^{\left(k-1\right)}}{\beta^{\left(k-1\right)}}\right)\right]\}-1,\\ \chi^{\left(k\right)} & = & \frac{{C_{B}^{\left(k\right)}}^{2}+1}{{\left(\mu^{\left(k\right)}\right)}^{2}}\frac{\Lambda^{\left(k-1\right)}}{\beta^{\left(k-1\right)}}\left(\frac{\Lambda^{\left(k-1\right)}}{\beta^{\left(k-1\right)}}-2\right)-\\ & & \frac{\Lambda^{\left(k-1\right)}}{{\left(\beta^{\left(k-1\right)}\right)}^{2}\mu^{\left(k\right)}}+e^{\left(k-1\right)}\frac{\Lambda^{\left(k\right)}}{\mu^{\left(k\right)}}\frac{{C_{B}^{\left(k\right)}}^{2}+1}{{\left(\mu^{\left(k\right)}\right)}^{2}},\\ e^{\left(k\right)} & = & \frac{1}{{\left(\beta^{\left(k\right)}\right)}^{2}}-\frac{1}{{\left(\beta^{\left(k\right)}\right)}^{3}}\sum_{l=1}^{k}\frac{\varrho^{\left(l\right)}}{R^{\left(k\right)}}\mu^{\left(l\right)}{C_{B}^{\left(l\right)}}^{2}. \end{array}$$

The Equation (24) corresponds to (21) in the case of G/G/1/N station: it defines how the variation in the interdeparture times of the class-*k* customers depends on the variations of the interarrival times of all classes that may influence the output of this class. The parameters of service time distributions are hidden in the coefficients of the equation.

Equations (18) and (24) taken together with (21) or (24) determine the input flow parameters for each class and each station, allowing us to analyze each station separately.

## 5. The SDN Switch

The SDN switches were modeled recently with the use of diffusion approximation in [47,48,57]. They considered the switch architecture discussed in [59] and simplified it to a single G/G/1/N station. They argued that since the input and output hardware of an SDN switch is fast, the main component of the switch introducing delay and therefore to be modeled, is the queue of packets waiting until the node identifies to which flow they belong and what output port they are to be sent. Suppose that the identification requires a linear search in a flow table with *K* entries, and *T* is the constant time to check one entry. Let ϵ be the probability that the router’s flow table does not contain the flow rule for a given packet; this will be discovered after going through all *K* positions, i.e., after time KT. In this case, the service time is constant, with zero variance. Otherwise, with probability (1−ϵ), the time to find the existing entry is uniformly distributed in [T,KT] and has a mean (K+1)T/2 and variance $\left(K^{2}-1\right)T^{2}/12$.

**Figure 21:**
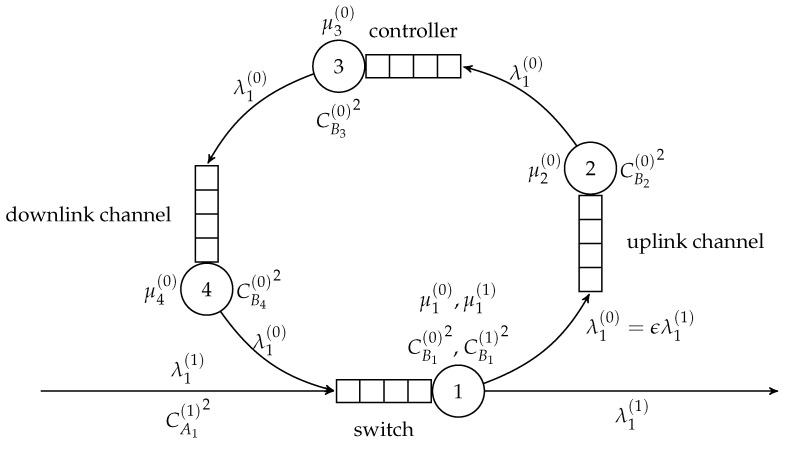
Model of SDN switch and its interactions with the SDN controller.

In the cited models, if a packet is not identified, it disappears. Here, we follow its way to the controller and its return to the switch via uplink and downlink channels as well as its second service in the switch as a priority customer, similarly as is done in [28], but considering transient behavior of the system and general interarrival and service time distributions. The model gives us a chance to see the delays introduced by the communication with the controller and priority service of returning packets. We may study the behavior of the system as a function of its parameters, such as speed of the switch, the controller and communication channels, and hit ratio for the identification of incoming packets. This system is presented in Figure 21. The model is composed of four service stations: the switch is a G/G/1/N/Priority station, and other stations are modeled as G/G/1/N. We use Equations (17), (18), (21), and (24) to separate the stations; the structure of the network is simple and these general formulas are thereby greatly simplified.

The length of the diffusion interval, i.e., the maximum size of the queue in the model, significantly affects the numerical solution time of the diffusion equation; the longer the interval, the greater the calculation time. To ease the calculations, we assume that the maximum volume of the switch buffer is N=50 packets when ϵ=0.2, but when ϵ=0.5, this means the congestion is higher and queues are longer, and we assume that N=100. The maximum size of other queues is N=20 packets.

**Figure 22:**
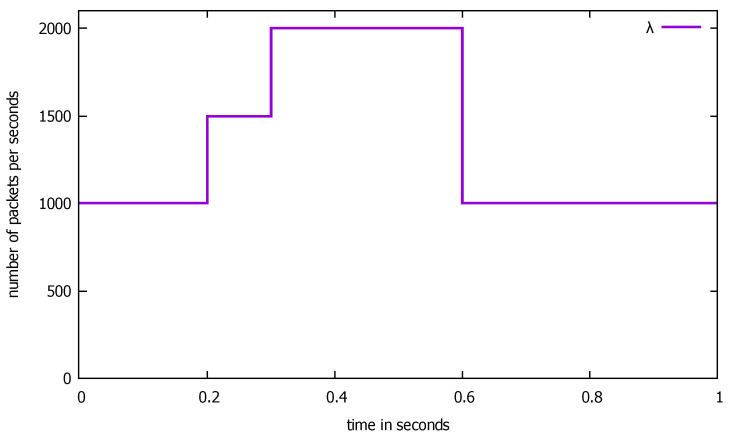
The input flow λ (packets per second) to the SDN switch, considered interval of 1 s.

In the numerical example below, the changes in the input flow are displayed in Figure 22. They cover an interval of 1 s. We used parameters K=950, $T=8\times 10^{-7}$ s (giving $\mu_{1}\approx 2630$ packets/s) to determine the distribution of service time at the switch and two values of the probability ϵ of missing a flow description. With this probability, a packet goes (only once) along the loop S2-S3-S4 and comes back to the switch S1 as a priority packet. We assumed for channels; i.e., stations S2, S4, $\mu_{2}^{\left(0\right)}=\mu_{4}^{\left(0\right)}=1000$ packets/s, and $\mu_{3}^{\left(0\right)}=1500$ packets/s for the controller. ϵ=0.2,0.5. The service time is either constant or uniformly distributed only for nonpriority packets; for priority packets, the distribution is uniform (ϵ=0 in this case).

The transient solution of diffusion equations is computed in time-intervals of the length 5 ms; i.e., we have 200 intervals with constant but different diffusion parameters following the state of the system. Inside an interval, diffusion parameters in single station models are constant; at the end of each interval, the Equations (17), (18), (21), and (24) furnish new traffic parameters for the the diffusion models at the next interval. The queue distributions at the end of an interval are used as initial conditions for the next one.

**Figure 23:**
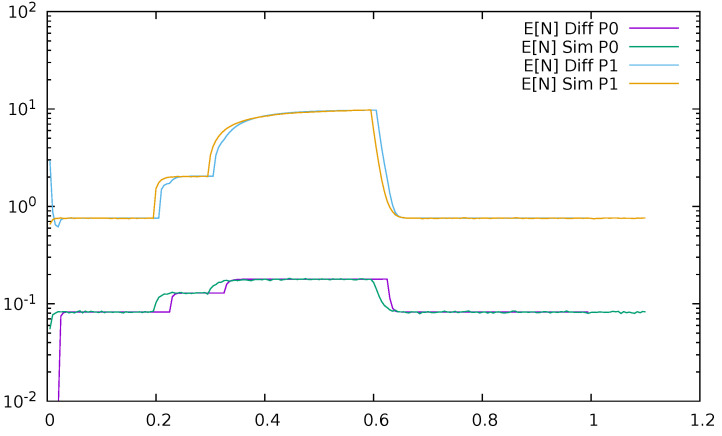
Mean queue length at the switch for priority (P0) and non—priority (P1) packets as a function of time, ϵ=0.2, diffusion, and simulation results.

**Figure 24:**
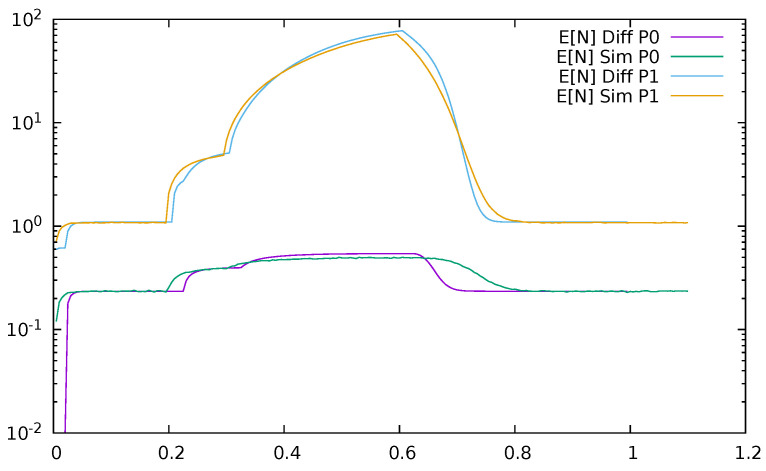
Mean queue length at the switch for priority (P0) and non—priority (P1) packets as a function of time, ϵ=0.5, diffusion and simulation results.

Below, a few figures illustrate the numerical results. Figure 23 displays the mean queue lengths at the switch for priority (P0) and non-priority (P1) packets as a function of time, reacting to the changes in the input traffic, for ϵ=0.2. We used a logarithmic scale to show together the results for both priority and non-priority classes, which have significantly different values. The simulation and diffusion approximation results are displayed together. Figure 24 presents similar results for ϵ=0.5. Comparing both figures, we see the impact of ϵ on the queues; its increase from 0.2 to 0.5 makes the switch maximum mean queue almost ten times longer.

**Figure 25:**
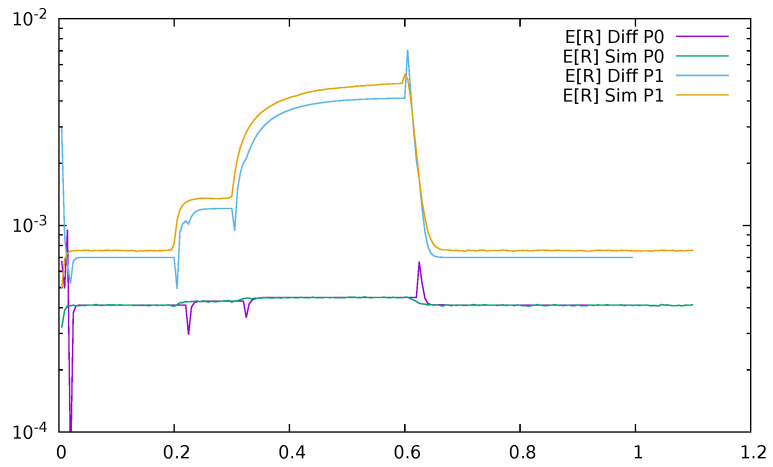
Mean response time at the switch as a function of time, ϵ=0.2, diffusion, and simulation results.

**Figure 26:**
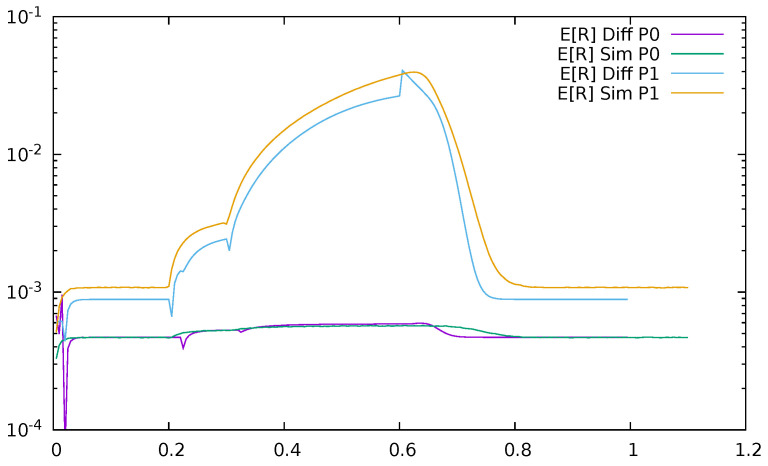
Mean response time at the switch as a function of time, ϵ=0.5, diffusion, and simulation results.

The same may be observed in Figure 25 and Figure 26, presenting mean response time of the switch as a function of time, for ϵ=0.2 and ϵ=0.5, diffusion, and simulation results. The change in ϵ greatly influences the delays. The spikes at the moments of traffic changes come from the fact that we used the Little’s formula E[R]=E[N]/λ, which is correct at steady-state analysis but approximate in the transient one, to obtain the mean response time.

**Figure 27:**
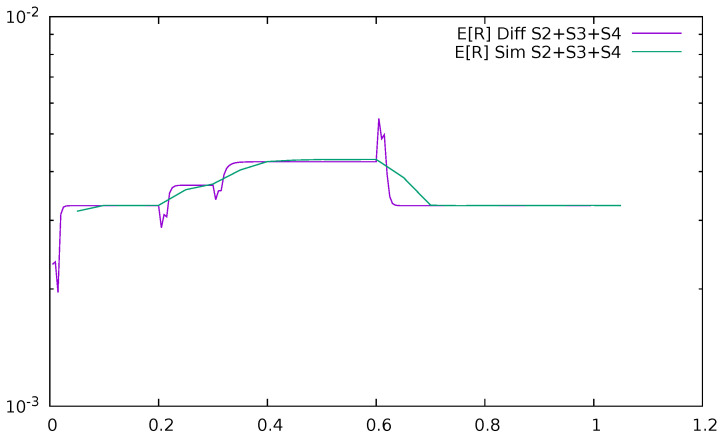
Mean delay introduced by the communication with the controller as a function of time, ϵ=0.2, diffusion, and simulation results.

**Figure 28:**
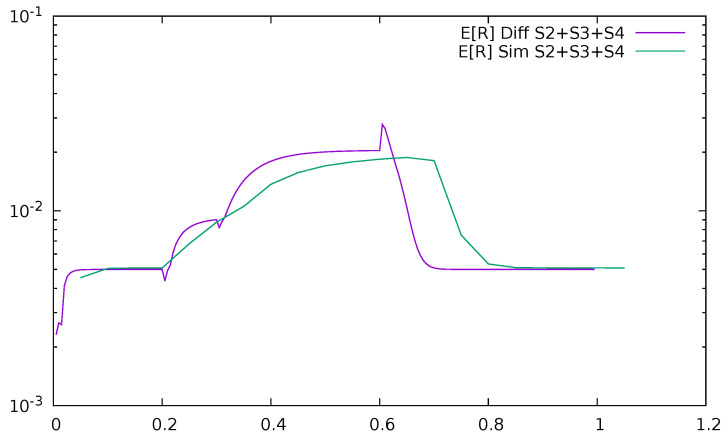
Mean delay introduced by the communication with the controller as a function of time, ϵ=0.5, diffusion, and simulation results.

Figure 27 and Figure 28 give the time-dependent mean delay introduced by the communication with the controller, i.e., the summary mean response time of uplink and downlink channels and the controller, $E\left[R_{2}\right]+E\left[R_{3}\right]+E\left[R_{4}\right]$, after which the packets of previously unrecognized destination come back to the switch together with their flow details. The entire mean response time of the system is

$$E\left[R\right]=E\left[R_{1}^{\left(1\right)}\right]+\left(1-\epsilon \right)\left(E\left[R_{2}\right]+E\left[R_{3}\right]+E\left[R_{4}\right]+E\left[R_{1}^{\left(0\right)}\right]\right).$$

and the pdf of the *R* is

$$f_{R}\left(x\right)=f_{R_{1}^{\left(1\right)}}\left(x\right)+\left(1-\epsilon \right)\left(f_{R_{2}}\left(x\right)*f_{R_{3}}\left(x\right)*f_{R_{4}}\left(x\right)*f_{R_{1}^{\left(0\right)}}\left(x\right)\right).$$

**Figure 29:**
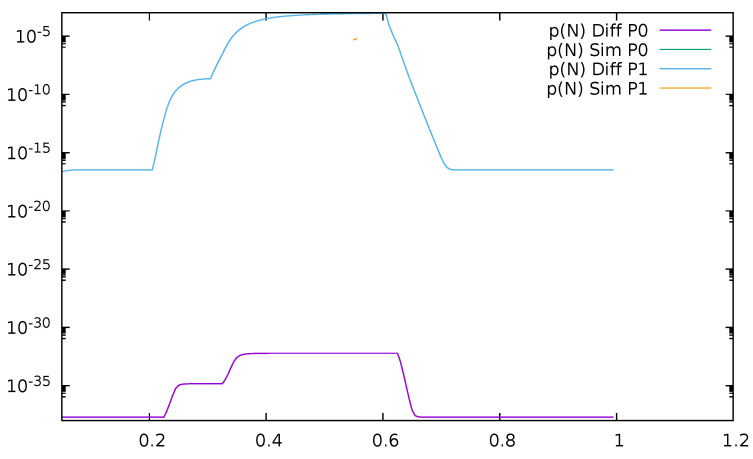
Loss probability due to the queue saturation as a function of time, ϵ=0.2, diffusion, and simulation results.

**Figure 30:**
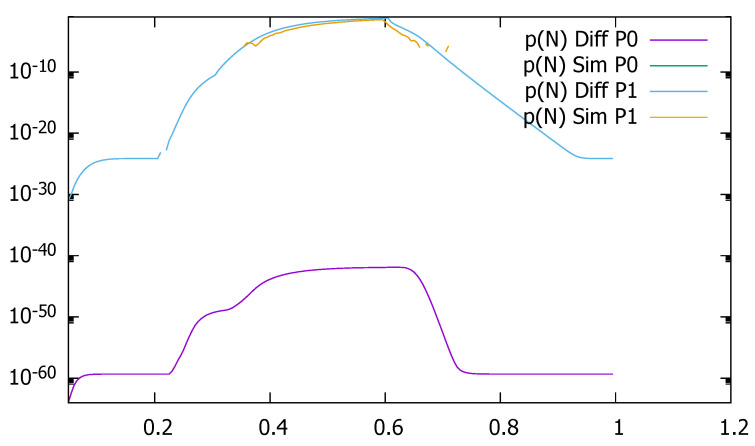
Loss probability due to the queue saturation as a function of time, ϵ=0.5, diffusion, and simulation results.

Figure 29 and Figure 30 present loss probability due to the queue saturation as a function of time, respectively, for ϵ=0.2 and ϵ=0.5, obtained by diffusion approximation and simulation. It is visible that 100,000 simulation runs are not enough to obtain reliable results; they are incomplete and mostly nonexistent, while diffusion approximation has no difficulties in modeling very small probability values.

The comparison of diffusion and simulation results gives us an estimation of errors introduced by the method, and we conclude that their size is acceptable in general. In addition, the dynamics of changes follows well the one observed in the simulation model. If the controller can change routing each 100 ms, the switch and a network of switches will operate in a transient regime for most of the time. Therefore, every performance evaluation or an optimization study should take a transient analysis into account. Diffusion approximation proves to be a convenient tool for this purpose.

Our original contributions are the following:

- Proposing the diffusion model of a multiclass G/G/1/N/Priority station, i.e., a station with general interarrival and service time distributions, limited buffer, and with preemptive-resume priority queues. Each class of customers has its specified priority level and its own parameters of the interarrival and service time distribution. Within one priority class, the scheduling is based on the FIFO algorithm. The model covers transient and steady-state analysis.
- Validation of this model by comparison with discrete-event simulation for various loads and interarrival and service time distributions, and discussion of errors;
- The model of an open network with any topology integrationg priority and FIFO service stations;
- A model of SDN switch exchanging packets with undetermined routing with SDN controller and validation of this model.

General distributions, priority classes, transient analysis, flexible topology, the form of results which is not restricted to mean values but giving the distributions, make the proposed model broader than the existing ones.

## 6. Conclusions

The article proposes a queueing model of G/G/1/N/preemptive-resume priority station serving customers with any number of priority classes. The main features of this model are general distributions of interarrival and service times and transient analysis of the queues. The results of a single station model were verified and validated in detail by comparison with simulations for various patterns of time-dependent traffic. In addition, the results of the ring switch–uplink–controller–downlink were verified with the simulations. In most cases, the approximation quality is very good, especially when the squared coefficient of variation of interarrival and service time distributions is close to one. The factors negatively affecting the approximation are: the increasing number of priority levels, because the results for a certain class depend on results (and errors) for all higher classes, and very large variances of interarrival and service time distributions. Furthermore, the results for the network are worse than for a single station: the errors of determination of variation of flows and the errors of dynamics prediction in intermediate stations (uplink, controller, downlink) bring additional errors into the switch model.

The model gives an insight into the performance of a priority service station. The impact of the utilization of the system on queue lengths and response times at various priority levels is visible. A network model integrating any number of G/G/1/N/preemptive-resume and G/G/1/N stations, both for the steady-state and transient regime, is presented and used to study the performances of SDN switch receiving a flow of packets with the intensity, which is frequently changing due to the decision SDN controller. The model includes the communication between switch and controller for packets belonging to flows unrecognized by the switch. It may be used to study the impact of the speed of switch, controller, and communication between them on performances of the SDN network, including such quality of service factors as delay, jitter, and losses. It also allows us to evaluate the effect of the hit ratio (probability that a packet belongs to a flow that is known to the switch) on the switch response time, the possible starvation problem on the lower priority level, and loss probabilities of packets. Another advantage of the diffusion approach is that it gives us the distributions of queues and delays, not only their mean values. It also means that we determine the probability that a packet is lost because of the saturation of the buffer. The obtained numerical results indicate that the transient regime may take a significant part of the total switch operation time; therefore, the diffusion approach to study transient periods is fully justified. In future work, we will focus on validating the model of the entire SDN network with any number of switches.

## Data Availability

Not applicable.
